# Promoting and Supporting Positive Conversations and Knowledge Mobilisation About Organ Donation in NHS Staff: a Hashtag “#” Series of Projects

**DOI:** 10.3389/ti.2025.15131

**Published:** 2025-09-09

**Authors:** Natalie L. Clark, Dorothy Coe, Hannah Gillespie, Marcus Diamond, Michael O’Malley, David Reaich, Caroline Wroe

**Affiliations:** ^1^ South Tees Hospitals NHS Foundation Trust, Middlesbrough, United Kingdom; ^2^ Newcastle-Upon-Tyne Hospitals NHS Foundation Trust, Newcastle upon Tyne, United Kingdom; ^3^ MIMA School of Art and Design, Teesside University, Middlesbrough, United Kingdom

**Keywords:** organ donation, opt-out legislation, national health service (NHS), United Kingdom, England

## Abstract

Implementation of the “soft” opt-out legislation in England has not had the desired impact in increasing the number of deceased donations and consent. The need for organs continues to be greater than the number of organs available, consent rates have fallen and organ donor registrations have stagnated. Introducing the legislation during the pandemic has had a profound effect with public awareness campaigns withheld, leaving a significant proportion of the population unaware of the change. Strategies to increase the public’s awareness and understanding of organ donation and the opt-out legislation are needed, as well as to encourage decision-making and sharing this with their families. We outline several “#” projects (#conversations, #options, #speak) with NHS staff to demonstrate how we can successfully utilise this specific population as trusted individuals and advocates to promote positive communications about organ donation and the opt-out legislation. NHS England is one of the biggest employers and most ethnically diverse across Europe. We know that NHS staff are more supportive, more aware and are more likely to have made an organ donation decision and had conversations with their families than the public. This places them in a unique and valuable position to lead positive conversations about organ donation.

## Introduction

Within the United Kingdom (UK), the number actively waiting for a transplant exceeds the number of organs available for transplantation, resulting in patients being removed from the list due to deteriorating health or from dying while waiting. In May 2020, the organ donation legislation in England changed from opt-in to “soft” opt-out [1]. This means consent to be an organ donor is assumed unless an individual explicitly registers their decision on the organ donor register (ODR) that they opt-out or they meet exclusion criteria whereby assumed consent cannot apply. With the “soft” opt-out system, a family member will still be consulted and can override the donor’s wishes in comparison to a “hard” opt-out system whereby a donor’s decision is the primary factor [2]. By June 2023, this legislation had been implemented across the UK, with Wales first implementing this in December 2015 [1].

The change was introduced with the intention to improve the number of available deceased donors, as has proven successful in countries like Spain [3, 4]. However, this change in legislation coincided with the COVID-19 pandemic which impacted adversely on organ donation internationally and overshadowed the planned public awareness campaigns, with the “Pass it on” slogan removed [5]. The impact of implementing the opt-out legislation within the UK is likely to have been overestimated whilst additional factors such as economic implications, the role of family members, public health initiatives, engagement with stakeholders, and training of healthcare professionals, have been underestimated [6–8]. Subsequently, a significant number of the population are unaware of the change and vulnerable to misinformation.

By 2023, consent rates across England, Scotland and Wales failed to recover beyond pre-pandemic levels, from 68.3%, 63.0% and 63.6% in April-June 2019 to 63.2%, 60.5% and 56.3% in April-June 2023, respectively [3]. The latest statistics from National Health Service Blood and Transplant (NHSBT) showed those registering an opt-in decision on the ODR stagnated from 27.7million registered in 2022 to 28.4million in 2025 [9]. The list of patients actively waiting for an organ transplant had started to decrease up until the COVID-19 pandemic, however the 2024/25 NHSBT activity report states there are 8,096 patients waiting [9], this figure being the highest seen in 15-year where approximately 8,000 were on the waiting list in 2009/10 [10].

### The Spanish Model of Organ Donation

Spain is recognised as the world leader for organ donation and transplantation, operating an opt-out legislation since 1979 [11]. It is estimated to have been approximately 10 years before the effects were seen [12]. However, the legislative change in isolation did not contribute to the country’s success and normalisation of organ donation within their society. The Spanish Model of Organ Donation consists of three components, a solid legislative framework, strong clinical leadership and a highly organised logistics framework [11, 13–15]. Factors constituting the model include simpler consent processes, access to more intensive care unit (ICU) beds, better resources, ensuring organ donation is embedded within the overall healthcare system, providing legal protection for organ donors, and greater education and training for healthcare professionals. Spain’s Model should be viewed as an international exemplar for deceased donation and could be replicated in other countries, like the UK, to improve deceased donation rates alongside the legislative changes.

**FIGURE 1:**
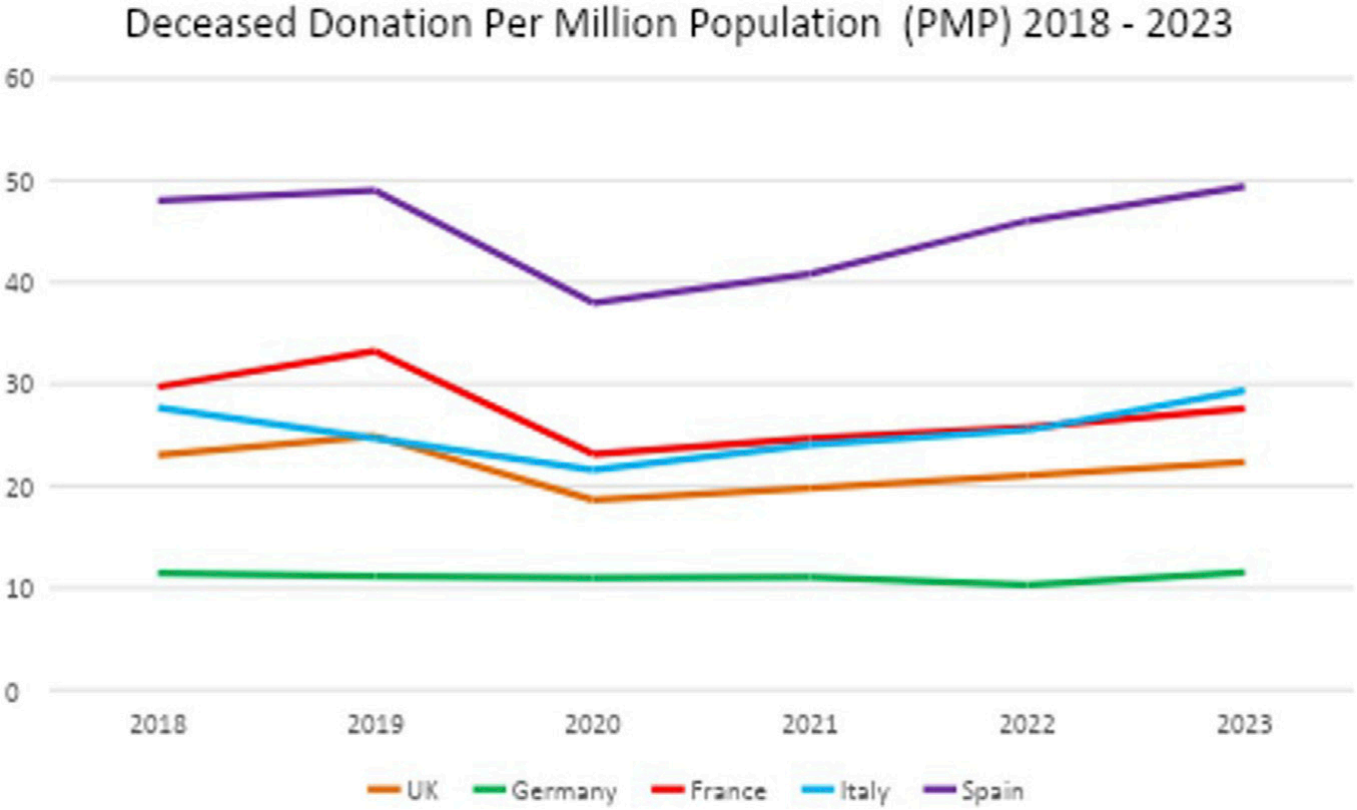
Deceased donation per million population (PMP) from 2018 to 2023 in the UK, Germany, France, Italy and Spain.

To understand the trends of deceased donation in the UK from 2018 to 2023 (inclusive), we used open access data from the International Registry in Organ Donation and Transplantation (IRODT) [16] and compared the rates of deceased donation against four countries of a similar population size (Spain, Germany, France, Italy) (Figure 1). Pre-pandemic (2018–2019), deceased donation per million population (PMP) rates were increasing across the UK, Spain and France. In 2020, UK, Spain, France and Italy all experienced a decline in deceased donation PMP rates, though they began to recover the following year.

However, by 2023 and compared to 2019, the UK (24.88 vs. 22.35 PMP) and France (33.25 vs. 27.63 PMP) had not returned to pre-pandemic deceased donation PMP rates, respectively. Germany deceased donation PMP rates stayed consistent throughout 2018–2023 at around 11 PMP. Whereas, both Spain and Italy had improved beyond their pre-pandemic rates. Italy similarly implemented an eight-step organ donation and transplantation programme [12, 17], heavily influenced by the Spanish Model, evidencing successful replicability. Recent figures from 2024 [16] suggest deceased donation PMP rates in the UK have fallen (20.37) whilst Spain remains the highest globally.

The “soft” opt-out legislation in England means an organ donor’s family are still consulted, as is the case for Spain. The NHSBT 2024/25 statistics highlight that the family are more likely to be supportive when a donor has expressed a decision to opt-in, with consent/authorisation at 87% versus when a decision was unknown at 48% [9]. Ethnicity is a known influencing factor on consent rates, with much lower family consent/authorisation rates (33%) from ethnic minority groups [1, 9]. In Spain, the number of refusals by family has progressively declined, one explanation being improved communication amongst families regarding sharing individual wishes in conjunction with an overall positive attitude towards donation within society [11, 18]. A priority within the UK is to encourage conversations with families and sharing organ donation decisions which can then increase consent to organ donation [11, 18, 19].

### The Unique and Valuable Position of NHS Staff

The National Health Service (NHS) is one of the biggest employers and most ethnically diverse across Europe, with over 1.5 million employees in NHS England as of January 2025 [20, 21]. Our objective is to demonstrate how we can successfully utilise this specific group of individuals to become organ donation advocates and lead positive conversations and foster constructive dialogue about organ donation, extending into our communities. We define a positive conversation about organ donation as one that is supportive, engages empathetic listening, and is respectful of one’s wishes [22].

In 2017, we conducted a pilot survey over 5-week in partnership with “ExtraLife” (Table 1). Our survey aimed to explore the views about and barriers to organ donation in the local workforce, including NHS staff based within two Trusts (one acute medical, one mental health). The questions were derived from the NHSBT Optimisa Survey of the General Population, 2013 [19]. We found NHS staff to be largely supportive of organ donation (96%) with the majority willing to donate their organs after death (90%). Approximately 74% of respondents were already registered to the ODR, 71% had shared their decision with someone else and 97% understood the importance of making their family members aware of their decision (Table 2; Supplementary Material S1)

**TABLE 1:** Summary of the ExtraLife survey and the three # projects (#conversations, #options, #speak).

Project	Target population	Objectives	Results	Impact
ExtraLife Survey *2017* Funded by the local organisations involved	Employees (n = 549) from Teesside working in the NHS (one mental health Trust and one acute medical Trust), and the local College, Football Club, Council and University	To explore views about and uncover barriers to organ donation in the local Teesside workforce To use the findings to develop workplace-based education to support organ donation	NHS staff were • The most supportive of organ donation (96%) • Most likely to be registered on the Organ Donor Register (ODR) (74%) • Most likely to agree on the importance of making their family aware of their decision (97%) However • 30% did not know how to put their name on the ODR • 25% did not have enough information to decide	NHS staff were identified as a large potential advocacy resource for organ donation Results were shared with transplant patient group and reviewed by Graphics Design students and lecturers at Teesside University to develop novel educational tools to support organ donation conversations (the #conversations short film)
#conversations short film *January to February 2019* Funded by ExtraLife, Principle Sounds and Northern Counties Kidney Research Fund	NHS staff working in one Acute Medical Trust in Teesside (n = 338) attending mandatory Basic Life Support (BLS) training	To create a short educational film that can be delivered to NHS staff during BLS training to promote positive conversations about organ donation To evaluate the effectiveness of the #conversations short film as an educational tool for NHS staff	Before watching the short film • 99% supported organ donation • 72% discussed organ donation with friends/family After watching the short film • Understanding of the need for organ donation (95%) and impact on family (94%) increased • 43% had talked to their colleagues about organ donation • 51% had talked to their family about organ donation • 42% were more supportive of organ donation	The #conversations short film promoted impactful and thought-provoking conversations about organ donation and was effectively delivered within BLS training with minimal additional resources
#options survey *July to December 2020* Funded by the Northern Counties Kidney Research Fund	NHS staff working in primary and secondary care, mental health, ambulance and community services in the North East and North Cumbria (n = 4986), and North Thames(n = 803)	To investigate the levels of awareness, support and action taken towards the new organ donation legislation in England, in NHS staff To understand what influences opinions (e.g., demographics, geographical location) To use the findings to support the development of educational resources around organ donation and the change in legislation for NHS staff	NHS staff were • More aware than the public (68% vs. 60%) • More supportive (83%) • 6x more likely to have had a conversations about organ donation • 3x more likely to already be on the ODR. The survey demonstrated higher levels of awareness and support across ethnic minority groups NHS staff also requested more information about • The process of organ donation • How relatives are informed • How to opt-out on behalf of loved ones • Storage of data and decisions • Understanding the rationale behind the legislative change • What has been communicated to the public and patients	Notably, NHS staff, compared to the general public, have taken more positive action in response to the change in legislation and were more likely to have had conversations with their family and friends There was a desire from NHS staff to find out more information Based on the findings, there is a unique opportunity to support NHS staff to be advocates and ambassadors for organ donation and the change in legislation
#speak focus groups *May 2024* Funded by the Northern Counties Kidney Research Fund	Participants of the #options survey who consented and provided their contact details to take part in future focus group work and attended focus group sessions (n = 14)	To conduct focus group discussions with NHS staff to review two educational resources, #conversations short film and an NHSBT resource To determine any educational gaps	NHS staff liked it when the videos • Had accessible language • Used real people to make it personal • Graphics were not too overwhelming NHS staff felt the videos • Missed emphasis improving quality of life • Should ensure statements were phrased appropriately • Should include how you can opt-in NHS staff felt a new resource could • Include a tangible message • Be a part of annual event • Include about how to overcome barriers • Empower families	The focus groups helped to understand how the #conversations short film can be adapted, upscaled and implemented more widely across other NHS Trusts delivering BLS training or in other similar settings A greater understanding of the specific information NHS staff (clinical and non-clinical) want to know about organ donation, following on from the #options survey Using these findings, we can develop an educational resource to further support the wider NHS workforce to be more knowledgeable about organ donation, and support conversations with their family, friends, colleagues and the wider communities

**TABLE 2:** Pilot survey findings of NHS staff based within one acute medical and one mental health NHS Trust in the North-East and North Cumbria to understand current attitudes and barriers towards organ donation.

Question	Yes (%)	No (%)	Unsure (%)
Do you support the principle of organ donation?	343 (96)	3 (1)	10 (3)
Would you be willing to consider giving your organs after death?	320 (90)	11 (3)	25 (7)
Have you put your name on the NHS ODR?	260 (74)	78 (22)	14 (4)
Have you told anyone that you have put your name on the NHS ODR?	246 (71)	69 (20)	30 (9)

	Very/quite important	Neither important/unimportant	Quite/very unimportant
How important do you think it is to tell those closest to you of your wishes about donating your organs after death?	344 (98)	4 (1)	2 (1)

Following this, we conducted a larger survey between July-December 2020 called #options [23], with approximately 6,000 NHS staff across NHS organisations (primary care, secondary care, mental health, ambulance, community services) in the North-East and North Cumbria and North Thames (Table 1). We aimed to explore NHS staff views of organ donation and the opt-out legislation, including awareness, support and action taken around the ODR and conversations with family and friends. We compared our findings to those of the public, evidencing NHS staff were much more likely to be aware and supportive of the legislative change. They were also three times more likely to have registered a decision on the ODR, whilst 75% of NHS staff had reported already having had conversations with their family, compared to 12% of the public. More importantly, we highlighted greater awareness and support in ethnic minority groups within NHS staff, though this group were more likely to request more information to improve their knowledge of organ donation and the opt-out legislation [24].

Through both surveys, we have established higher levels of support for and awareness of organ donation amongst NHS staff, irrespective of whether they are directly/indirectly involved in organ donation and transplantation. Notably, the #options survey [23] was the largest survey of NHS staff to evaluate awareness and support for organ donation, including staff working across a variety of healthcare settings.

Due to the size of the NHS workforce, it is likely most families have a member, or know someone, who works for the NHS [13]. This places those individuals in a unique and valuable position as a trusted individual and advocate, to lead positive conversations about organ donation. These conversations have the scope to extend beyond healthcare settings into wider communities. Interventions, specifically for NHS staff, aiming to improve communication about organ donation are needed to support the sharing of accurate information (e.g., processes, reasons behind the change, family involvement [24]) and challenge any myths and misconceptions.

### Educational Resources for NHS Staff

We know from the #options survey that NHS staff, in clinical and non-clinical roles and those without direct involvement in organ donation, have expressed a desire for more information about organ donation and the opt-out legislation [24].

Educational interventions have previously proved effective in improving organ donation knowledge amongst healthcare professionals [25]. These interventions also aimed to increase organ donor numbers, improve identification of potential organ donors, improve referral processes, increase education, and provide extra support to families. However, the interventions were mostly delivered to and tailored for ICU staff or Specialist Nurses in Organ Donation (SNODs) who, due to the nature of their roles, we would already anticipate being organ donation advocates. Providing training and educating beyond these roles is of paramount importance to promote a collaborative approach, improve communication between healthcare professionals, patients and their family, and provide adequate support to families. This will further encourage all staff within an NHS setting to become more knowledgeable and aware.

In May 2024, we conducted the #speak project using two focus groups with 14 NHS staff from the North-East and North Cumbria, to explore gaps in educational resources for the wider NHS staff workforce, what they felt was missing and how this should be delivered (Tables 1, 3). There were several suggestions regarding the content that should be included. Examples being, providing clarity around the new assumed consent, overcoming barriers, clarifying eligibility criteria and the family involvement. As the target audience for these educational resources would be NHS staff, the focus group felt they could include more facts and statistics compared to if this was aimed at the public.

**TABLE 3:** Focus group summary of gaps in educational resources for the wider NHS staff workforce, what they felt was missing and how this should be delivered.

Theme	Recommendations
Format	• Technological poverty, provide information in other ways • Range of different delivery methods because it’s not a one size fits all • Use different approaches for different target audiences • More general education • If you’re tailoring it towards NHS staff you can be a bit sort of more numbery and statistics • Trying to be positive in what can be quite an awful time • Any big existing myths exist that would make someone not want to donate - a Q&A document “busting” these myths • It’s about managing expectations
Delivery	• Avoid making this another mandatory e-Learning course • Mandatory training can be seen as a tick box exercise and we definitely don't want this to be that • Screensavers on the Intranet • As a member of the NHS staff you are in a unique position of understanding that the work that we do impacts the work you do now but it could also impact the future and showing the importance the uniqueness and the value of that position to encourage people to engage with it which I think is a totally different offer than a mandatory training
Process (including consent, eligibility etc.)	• Personally confused around presumed consent then we’re still getting consent • What happens in terms of getting the body back for funeral arrangements • Process is what actually happens to somebody’s body • Who owns your body, that dark side of is it the state or do you own your body up until that point of your last breath • Making sure that everybody is aware of what that eligibility criteria is and not just the basics
Family	• Does your family know your views, whether it's opt-in or opt-out • Empowering families to ask if organ donation is possible for your family member - I know my family members wishes • Knowing your bit won't be affected knowing you can still be with your loved one • Nothing being disruptive in the most disruptive time

### A Short Film to Promote Conversations

Basic Life Support (BLS) training is mandated in the UK by the Resuscitation Council that all NHS clinical staff must undergo BLS training, with annual refresher sessions to maintain knowledge and skills [26]. Non-clinical staff within the NHS are expected to have the same BLS skillset as a lay person.

In a previous project (#conversations) [27], we explored how a short film could be embedded within in-person BLS training at one NHS Trust, using existing resources, and delivered to staff to promote conversations about organ donation (Table 1). We developed the film in collaboration with families from the North-East and North Cumbria who had direct experience of organ donation, placing emphasis on the personal narrative, and the ease and normality of having conversations around organ donation in everyday life [28–32]. Sharing personal stories of organ donation have proven to significantly impact registrations in the UK [1]. A bespoke soundtrack was developed by Principle Sounds^©^, supporting the message through emotional engagement [33]. The film was reviewed by families, a staff focus group and BLS trainers for feedback.

Over a 6-week period, the film was delivered to 228 NHS staff. We collected a baseline questionnaire of opinions towards organ donation [19] and followed-up NHS staff 5 days after attending BLS training and watching the film to assess impact.

Like our previously reported #options survey findings [23], the NHS staff demonstrated high levels of support for organ donation (99%) and were more likely to have shared their views towards organ donation with their family or friends (72%). Comparative data from NHSBT’s attitudinal survey in June 2020 showed only 49% of the public have had a conversation about organ donation, with even fewer saying they have shared their decision. Those completing the follow-up questionnaire felt that the film increased their understanding of the need for organ donation (95%) and the impact it has on families (94%). A further 43% of staff had talked to their colleagues and 51% had talked to their family about organ donation. Support for organ donation did not decrease after watching the film, with 42% even more supportive. Despite the widespread support at the time of the study, 25% were not comfortable with the proposed legislative change, 16% were unsure and 9% were unsupportive.

During the recent #speak project we utilised the focus groups to gather feedback about the #conversations film. With consent, we recorded group discussions, anonymised the recordings and analysed the data. We aimed to generate suggestions for improvement (Table 4). NHS staff particularly liked that the film led them “*on a journey*” to “*seeing some good results*” using real-life people and “*hearing real life stories*” to “*make it more personal*” and “*really impactful*”. Suggestions of improvements included, amending the volume of the music as they felt it was “*slightly too loud*” and at times, the way in which the graphics was used was sometimes perceived as being “*too much*” and this made it difficult to focus on the voiceover. Though #conversations was intended to be emotionally thought-provoking, some felt this made it a “*bit harder to tune into the rest of it because your arousal level is quite high*”. However, it was reiterated, “*that’s what’s needed*”.

**TABLE 4:** Focus groups summary of the short film (#conversations) and suggestions for improvement.

	#conversations
Positives	• Real people would make it more personal • Great hearing real life stories • Addition of the mum at the end was a really nice touch • Led me on a journey to why it was a good idea and seeing some good results • Appealed to my compassionate side • Lends to your emotional side • Definitely hits the spot, it’s very emotive, that’s what’s needed • Having the lady talking through her story with her son and the picture of her son was really impactful • The son being so young it was saying that everybody could have the conversation it’s not just for an older audience • What kind of impact that has and how everyday things that you do take for granted are special
Suggested improvements and recommendations	• Music slightly too loud • I felt like it was too much, I couldn't focus • Having everything all in one go is a bit too much for me • I felt quite immediately sort of moved then I don't know whether that makes it a bit harder to tune into the rest of it your arousal level is quite high • Need to be left with a tangible message – those are the conversations that people will lastingly have with their family • If you think more people can do it or everybody can do it there’s less personal onus on you to do, you’re in a very unique and privileged position • Ongoing thing rather than a thing that’s done and then that’s it • World awareness day - awareness stand, pens and posters, advertisements, people can go to the stand, walking past and having a look, having it annually pops up and you can create a buzz around it

NHS staff summarised that any new film, it should finish with a “*tangible message*”, and it is this message “*that people will lastingly have with their family*”. They also suggested that the content should aim to empower families, doing so will put them in position to “*ask if organ donation is possible for [their] family member*” as they will “*know [their] family members wishes*”. Ultimately, they agreed that “*there’s never a good time to talk about it so the conversations that we have earlier on the better*”. One NHS staff member suggested that this needed to be “*a regular ongoing thing*”, rather than something that is done once and then forgotten but not overdone “*to the point where people get desensitised*”, such as delivering any resources around significant awareness events (e.g., Organ Donation Week).

## Summary

Implementation of the opt-out legislation in England has not had the desired impact on increasing the number of potential eligible donors, consent rates and deceased donation. Introducing the legislation during the COVID-19 pandemic has undoubtedly had a profound effect and it is difficult to disentangle the true response to the change from the lasting effects of the pandemic. However, it is apparent that an opt-out legislation in isolation will not be effective in increasing the number of the opt-in registrations and consent. Instead, the UK would benefit from supporting an organ donation and transplantation programme that is embedded within society to improve public trust and confidence in deceased organ donation systems [3], as we have successfully seen in Spain and Italy [11–14, 17]. Currently, the public’s awareness and understanding of organ donation and the opt-out legislation is low, as is the number of individuals who have made a decision and shared this with their families. Increasing public awareness, understanding and support would be paramount within the programme.

We have outlined several projects with NHS staff from a variety of NHS organisations, with the film (#conversations) being delivered to and focus group (#speak) work specifically being with NHS staff from the North-East and North Cumbria. These findings are promising, demonstrating how we could work with this specific population to promote positive communications about organ donation and the opt-out legislation. There is a recognised need to involve NHS staff outside of those with direct involvement in organ donation and transplantation to maximise opportunities for deceased donation [34]. To effectively do so, we need to ensure that the wider NHS workforce is appropriately educated using tailored resources which will help them initiate conversations and provide support.

We must develop effective strategies to empower individuals to make their decision in life and share this with their families to reduce the number of family refusals. Doing so will begin to improve organ donation rates, and both save and improve the quality of lives of individuals actively waiting for a transplant. This could be done through revising and relaunching public health campaigns as some evidence suggests [34].

To further demonstrate the effectiveness of such resources, a consistent and systematic approach is needed, including standardising implementation across settings, clear outcome measures and appropriate data collection methods to evaluate impact reliably. We propose updating the film first used in #conversations by incorporating the feedback from the #speak focus groups and literature post-legislative change. The aim being to disseminate as part of a public health campaign that can be evaluated within a regional pilot study and funded by a partnership between the Department of Health and Social Care, NHSBT and local Integrated Care Boards. This will be evaluated within a regional pilot study. To evaluate impact, we will gather pre-/post-test regional data from NHSBT on ODR registrations, deceased donation consent rates, and transplant activity, further evaluating how impact varies across demographics (e.g., age, ethnicity) and geographical location, these factors being known to influence organ donation consent rates [9].

## Data Availability

The raw data supporting the conclusions of this article will be made available by the authors, upon reasonable request to the corresponding author.

## Abbreviations

BLS, Basic Life Support; ICU, Intensive Care Unit; IRODT, International Registry in Organ Donation and Transplantation; NHS, National Health Service; NHSBT, National Health Service Blood and Transplant; ODR, organ donor register; PMP, per million population; SNOD, Specialist Nurses-Organ Donation; UK, United Kingdom.
