# Supplementary material for: Promoting and Supporting Positive Conversations and Knowledge Mobilisation About Organ Donation in NHS Staff: a Hashtag “#” Series of Projects
Source: Transpl Int. 2025 Sep 9;38:15131. doi: 10.3389/ti.2025.15131 (PMC12456233; doi:10.3389/ti.2025.15131)
Supplement: Supplementary file 1 [file DataSheet1.docx]

**Supplementary material 1** Additional pilot survey findings of NHS staff based within one acute medical and one mental health NHS Trust (combined) in the North-East and North Cumbria to understand current attitudes and barriers towards organ donation.

|  | Strongly agree  (%) | Agree  (%) | Neither agree/disagree  (%) | Disagree  (%) | Strongly disagree (%) |
| --- | --- | --- | --- | --- | --- |
| I didn't know there was an NHS ODR | 7 (2) | 10 (3) | 17 (5) | 61 (19) | 227 (70) |
| I have a donor card and I didn't know I needed to register as well | 17 (5) | 59 (18) | 56 (17) | 61(19) | 128 (40) |
| I don't know how to put my name on the NHS ODR | 15 (5) | 51 (16) | 40 (13) | 69 (22) | 145 (45) |
| I don't have enough information about the register to make a decision | 11 (3) | 30 (9) | 48 (15) | 70 (22) | 162 (50) |
| I haven't got around to it yet | 17 (5) | 32 (10) | 48 (15) | 56 (18) | 166 (52) |
| It's too much hassle to fill the form in | 0 (0) | 3 (1) | 54 (17) | 84 (27) | 177 (55) |
| I don't need to make this decision until I am older | 3 (1) | 5 (2) | 21 (7) | 73 (23) | 218 (68) |
| I might change my mind and I don't know if you can take your name off it | 8 (3) | 26 (8) | 52 (16) | 63 (20) | 169 (53) |
| Organ donation is against my religion | 0 (0) | 0 (0) | 19 (6) | 44 (14) | 254 (80) |
| Organ donation is against my culture | 0 (0) | 0 (0) | 16 (5) | 41 (13) | 259 (82) |
| I don't know what my religion says about organ donation | 11 (3) | 27 (9) | 76 (24) | 29 (9) | 172 (55) |
| I don't know what my culture says about organ donation | 7 (2) | 10 (3) | 56 (18) | 42 (13) | 198 (63) |
| I am too old so my organs won't be of any use | 1 (0.3) | 1 (0.3) | 19 (6) | 91 (29) | 204 (65) |
| I have a medical condition which means my organs won't be able to be used | 3 (1) | 8 (3) | 35 (11) | 78 (25) | 193 (61) |
| I don't think I would qualify to be an organ donor | 1 (0.3) | 6 (2) | 30 (10) | 81 (26) | 197 (63) |
| I need to make a decision about becoming an organ donor so those closest to me don't have to when I die | 187 (58) | 95 (30) | 14 (4) | 12 (4) | 12 (4) |
| I trust my family to do the right thing if the worst happens and they need to decide whether to donate my organs | 134 (42) | 100 (31) | 35 (11) | 29 (9) | 22 (7) |
| I would want to consult with my family before putting my name on the NHS ODR | 38 (12) | 66 (21) | 46 (14) | 81 (25) | 88 (28) |
| I want my family to know how I feel about becoming an organ donor before I die | 182 (57) | 110 (35) | 11 (3) | 4 (1) | 10 (3) |
| I don't like the idea of talking to my family about organ donation | 11 (3) | 19 (6) | 38 (12) | 86 (27) | 163 (52) |
| I worry that my family will not approve of organ donation | 8 (3) | 14 (4) | 28 (9) | 105 (33) | 164 (51) |
| I worry that I might not really be dead when my organs are taken | 6 (2) | 31 (10) | 22 (7) | 68 (21) | 192 (60) |
| I don't like the idea of being cut up after death | 13 (4) | 42 (13) | 33 (10) | 73 (23) | 158 (50) |
| I don't want to think about my death | 30 (9) | 70 (22) | 54 (17) | 63 (20) | 99 (31) |
| I am happy to donate my organs when I die as I will no longer have any use for them | 212 (66) | 62 (19) | 23 (7) | 4 (1) | 18 (6) |
| I want my body to be whole when it is buried or cremated | 7 (2) | 9 (3) | 79 (25) | 82 (26) | 142 (45) |
| I don't want my body to be disfigured when my family sees it for the last time | 45 (14) | 72 (23) | 64 (20) | 51 (16) | 84 (27) |
| I worry about being viewed as an organ donor rather than a patient | 11 (3) | 44 (14) | 50 (16) | 98 (31) | 112 (36) |
| I trust doctors to always do what is best for their patients | 110 (35) | 143 (45) | 36 (11) | 15 (5) | 12 (4) |
| I worry that doctors may not do their best to save my life so they can take my organs | 5 (2) | 18 (6) | 38 (12) | 108 (34) | 148 (47) |
| If you would accept an organ transplant you must be prepared to be an organ donor | 125 (39) | 81 (25) | 54 (17) | 45 (14) | 15 (5) |
| Being on the NHS ODR should be automatic, unless you say you object | 153 (48) | 87 (27) | 27 (8) | 32 (10) | 21 (7) |
| Families should have the right to overrule a person's decision about organ donation after their death | 10 (3) | 14 (4) | 26 (8) | 107 (33) | 163 (51) |

**Supplementary material 2** Capsule Sentence Summary

Our paper describes projects that demonstrate how NHS staff can be successfully used as trusted individuals and advocates to promote positive communications about organ donation and sharing your organ donation decision.
